# Moving Domain Computational Fluid Dynamics to Interface with an Embryonic Model of Cardiac Morphogenesis

**DOI:** 10.1371/journal.pone.0072924

**Published:** 2013-08-23

**Authors:** Juhyun Lee, Mahdi Esmaily Moghadam, Ethan Kung, Hung Cao, Tyler Beebe, Yury Miller, Beth L. Roman, Ching-Ling Lien, Neil C. Chi, Alison L. Marsden, Tzung K. Hsiai

**Affiliations:** 1 Department of Biomedical Engineering, University of Southern California, Los Angeles, California, United States of America; 2 Department of Bioengineering, University of California Los Angeles, Los Angeles, California, United States of America; 3 Department of Mechanical and Aerospace Engineering, University of California San Diego, La Jolla, California, United States of America; 4 Division of Cardiology, Department of Medicine, School of Medicine, University of California San Diego, La Jolla, California, United States of America; 5 Department of Biological Sciences, University of Pittsburgh, Pittsburgh, Pennsylvania, United States of America; 6 Children’s Hospital Los Angeles, Los Angeles, California, United States of America; 7 Department of Surgery, Keck School of Medicine, University of Southern California, Los Angeles, California, United States of America; 8 Division of Cardiology, Department of Medicine, School of Medicine, University of California Los Angeles, Los Angeles, California, United States of America; Mayo Clinic, United States of America

## Abstract

Peristaltic contraction of the embryonic heart tube produces time- and spatial-varying wall shear stress (WSS) and pressure gradients (∇*P*) across the atrioventricular (AV) canal. Zebrafish (*Danio rerio*) are a genetically tractable system to investigate cardiac morphogenesis. The use of *Tg*(*fli1a:EGFP*)^*y1*^ transgenic embryos allowed for delineation and two-dimensional reconstruction of the endocardium. This time-varying wall motion was then prescribed in a two-dimensional moving domain computational fluid dynamics (CFD) model, providing new insights into spatial and temporal variations in WSS and ∇*P* during cardiac development. The CFD simulations were validated with particle image velocimetry (PIV) across the atrioventricular (AV) canal, revealing an increase in both velocities and heart rates, but a decrease in the duration of atrial systole from early to later stages. At 20-30 hours post fertilization (hpf), simulation results revealed bidirectional WSS across the AV canal in the heart tube in response to peristaltic motion of the wall. At 40-50 hpf, the tube structure undergoes cardiac looping, accompanied by a nearly 3-fold increase in WSS magnitude. At 110-120 hpf, distinct AV valve, atrium, ventricle, and bulbus arteriosus form, accompanied by incremental increases in both WSS magnitude and ∇*P*, but a decrease in bi-directional flow. Laminar flow develops across the AV canal at 20-30 hpf, and persists at 110-120 hpf. Reynolds numbers at the AV canal increase from 0.07±0.03 at 20-30 hpf to 0.23±0.07 at 110-120 hpf (*p*< 0.05, n=6), whereas Womersley numbers remain relatively unchanged from 0.11 to 0.13. Our moving domain simulations highlights hemodynamic changes in relation to cardiac morphogenesis; thereby, providing a 2-D quantitative approach to complement imaging analysis.

## Introduction

Hemodynamics has a significant impact on cardiac development [1]. Currently, assessment of mammalian mechano-transduction underlying intracardiac morphogenesis is hampered by the complexity of surrounding internal organ systems, coupled with a prolonged duration of development. Embryonic zebrafish (*Danio rerio*) is a genetically tractable model for investigating cardiac morphogenesis. Its transparency and short developmental time enables imaging and high throughput analysis of various developmental stages.

Fluid shear stress generated by circulating blood is intimately linked with cardiac morphogenesis. The shear forces impart mechano-signals to up-regulate developmental genes, with implications in endocardial cushion and atrioventricular (AV) valvular formation [1–4]. The exposure of the endocardium to shear forces also transmits mechano-signals to the myocardium [5,6], with implications in cardiac looping and trabeculation [6,7]. Clinically, developmental defects in the atrioventricular (AV) valve result in flow regurgitation, while the absence of an endocardial cushion results in AV septal defects in patients with congenital heart disease (CHD) [8]. Thus, hemodynamic analyses and interpretation of cardiac morphological changes are both clinically and developmentally significant.

Computational fluid dynamics (CFD) has been widely applied to simulate blood flow, to facilitate clinical decision-making, and to study the progression of cardiovascular diseases [9,10]. Despite a few studies about aortic arch morphogenesis and vascular morphogenesis [11–15], there is a paucity of literature in developmental embryonic zebrafish cardiac model reconstruction and application of CFD codes to establish quantitative analysis underlying morphological changes during development [16]. One report did directly measure intracardiac pressure in the adult zebrafish [17], however the technique is invasive, causing local flow disturbances when applied to the embryonic circulation. Indirect measurement of intracardiac shear stress requires high-resolution images for establishing the regions of interest, and for calculating fluid shear stress [1,2]. Particle image velocimetry (PIV) has been widely used to map the velocity vector fields because of its high speed data processing and not intrusive technique [18]. However, PIV is an optical technique, providing primarily 2D information, and with limited utility for three dimensional analysis capturing wall shear stress [19].

In this study, we sought to develop a two dimensional moving domain CFD model to model the progression of cardiac development. Segmentation of fluorescent microscope-generated image data provided boundary conditions to prescribe wall motion in the simulations. Endocardial boundaries were well demarcated between 20–30 hours post fertilization (hpf) and 110-120 hpf in the zebrafish embryos. Time-dependent CFD with moving boundaries was performed to provide biomechanical parameters relevant to development; namely, wall shear stresses (WSS) at the AV canal and pressure gradients (∇P) between the atrium and ventricle during embryonic zebrafish cardiac morphogenesis. Hemodynamic changes often precede and are correlated with morphogenesis [1,2,6]. Our computational approach, coupled with imaging of green fluorescent protein (GFP)-labeled transgenic zebrafish embryos, allowed for delineating the time-varying endocardium boundaries and for tracking blood particles to quantify the hemodynamic milieu in which cardiac looping, atrioventricular (AV) valve and ventricular development occur. The moving domain CFD simulations establish a quantitative baseline, enabling hemodynamic analyses underlying mechano-transduction and heart morphogenesis.

## Methods

### Zebrafish maintenance and embryos collection

**Figure 1:**
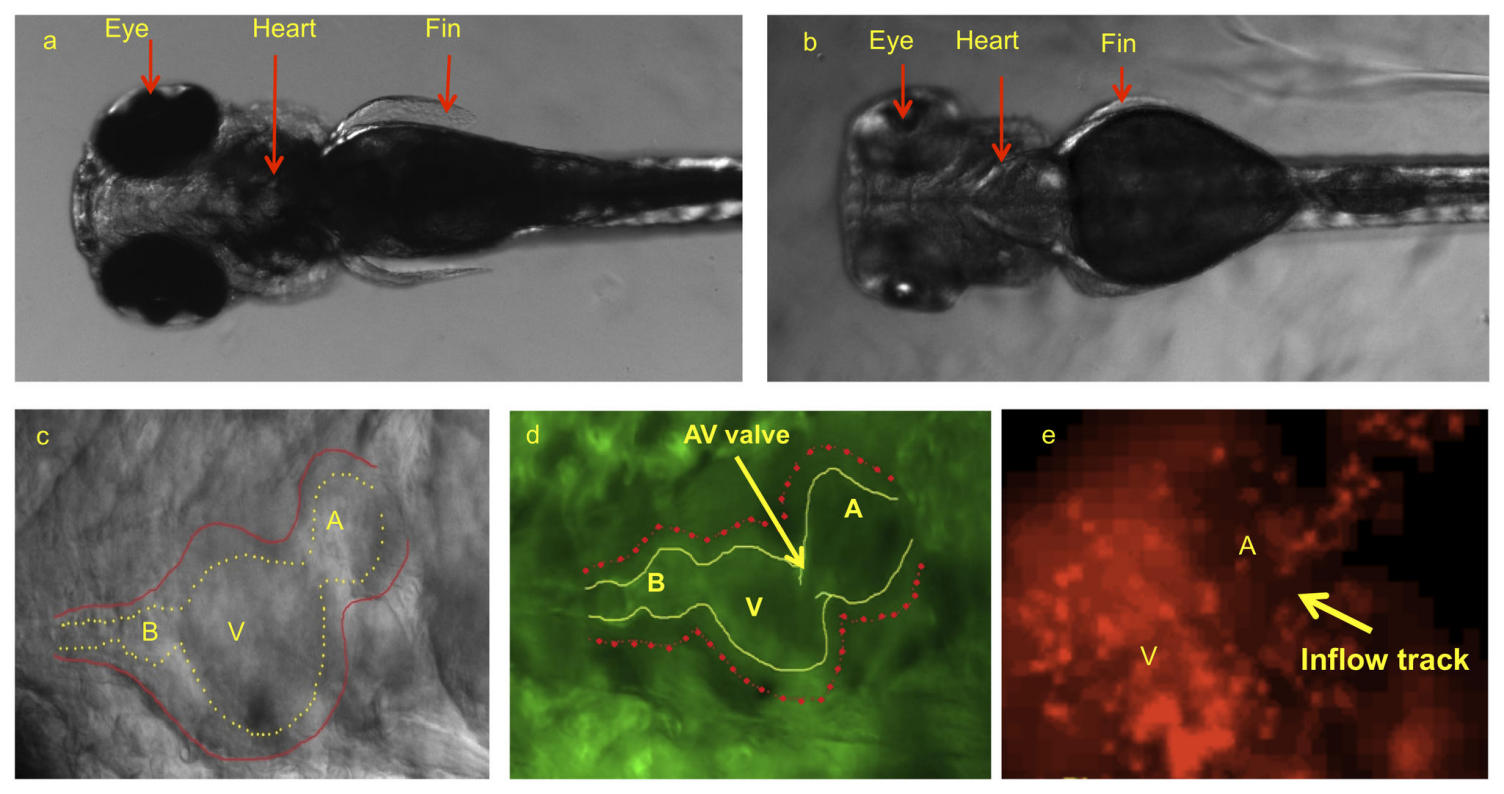
Dorsal view of zebrafish embryos illustrated cardiac system prior to and after pigmentation removal. (a) Pigmentation opacifies internal organ systems at 120 hpf. (b) Treatments with PTU beginning at 10 hpf prevented pigment formation, allowing for clear organ visualization. (c) PTU-treated zebrafish heart observed under bright field microscope at 120 hpf. Despite visualization of outer wall, inner wall is poorly demarcated. (d) The use of transgenic *Tg*(fli1a:EGFP)*y1* embryos allows for clear delineation of inner wall for reconstructing the CFD model. (e) *Tg(gata1:dsRed)sd2* transgenic zebrafish allows to visualize the blood particle in order to take velocity by particle tracking technique. A, atrium; V, ventricle; B, bulbus arteriosus.

Adult *Tg*(*fli1a:EGFP*)^*y1*^ *and* *Tg(gata1:dsRed)sd2* transgenic zebrafish were raised in the aquarium system (Aquaneering Inc. San Diego, CA) located in the vivarium of University of Southern California (USC). All the experiments were performed in compliance with the approval of USC Institutional Animal Care and Use Committee (IACUC) protocol (Zebrafish IACUC Protocol number: 11767). These fish were maintained with filtered fresh water under 14 hours of incandescent light and 10 hours of dark conditions [20]. The *fli1a* promoter-driven enhanced green fluorescent protein (GFP) is expressed predominantly in vascular endothelial and endocardial cells. The gata1, the red fluorescent protein, is an erythroid-specific transcription factor. The crossline embryos between *Tg*(*fli1a:EGFP*)^*y1*^ *and* *Tg(gata1:dsRed)sd2* were transferred to a petri dish and incubated in 28.5^o^C [2]. To maintain transparency of zebrafish embryos, embryo medium was supplemented with 0.003% phenylthiourea (PTU) to suppress pigment formation at 10 hpf (Figure 1a-b) [21].

### In vivo Imaging

**Figure 2:**
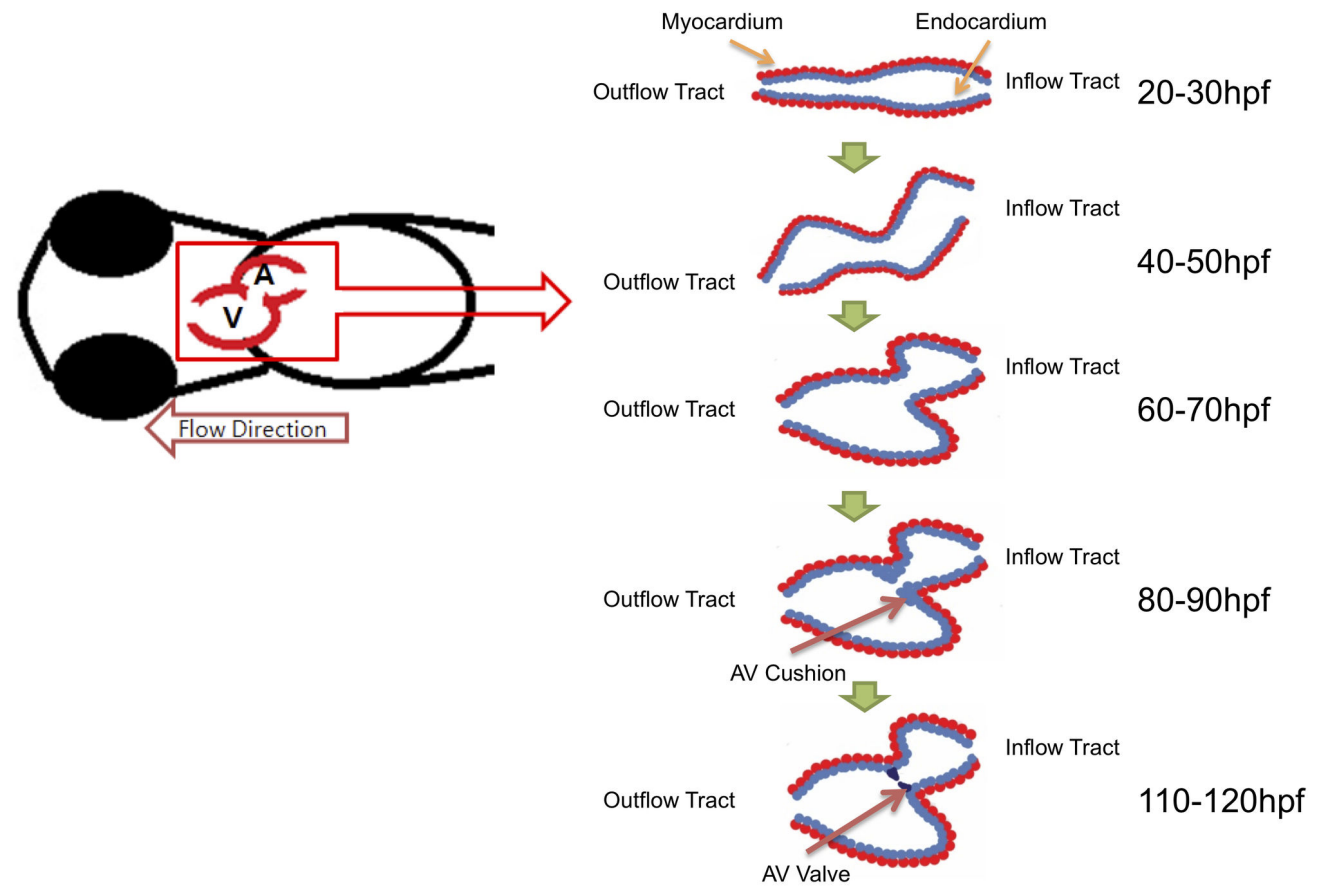
Schematic drawing of cardiac development in each stages. At the early embryonic stage, heart shape is a tube-like structure. At about 20 hours, zebrafish heart is undergoing looping. After completion of cardiac looping, AV cushion starts to develop into a AV valve.

At 30 hpf, 6 zebrafish embryos were anesthetized in 0.05% tricaine mesylate in compliance with the IACUC protocol [1,22]. Next, they were transferred onto the petri dish containing 1% low-melt agarosegel (UltraPure™ Low Melting Point Agarose, Invitrogen^TM^, Carlsbad, CA, USA) for immobilization. An inverted epifluorescence microscope (IX71, Olympus, Tokyo, Japan) with 20x lens, QIClick^TM^ digital CCD camera (Surrey, BC, Canada) and Qcapture Pro software (Surrey, BC, Canada) was used to visualize circulating red blood cells in the embryonic heart for velocity measurement. Moving endocardial boundaries were acquired with GFP-labeled endothelial cells (Figure 1c-d). The experiment was repeated with 6 embryos (n=6). After recording videos for 20-30 hpf samples, embryos were returned to the incubator at 28.5^o^C. This procedure was repeated every 20 hours until the zebrafish reached 110-120 hpf, at which time a morphological heart has developed. The individual cardiac developmental stages are illustrated in Figure 2 (Video S1).

### Velocity, viscosity, and density for simulations

Inlet boundary velocities were acquired from the red fluorescent video (Figure 1e, Video S2), and the movement of red blood cells was tracked by a particle image velocimetry (PIV) code written in Matlab (Mathworks, Natick, MA). Two-dimensional cross-correlations based on fast Fourier transform (FFT) were used in the PIV code to calculate the velocity vectors of the red blood cells. A multi-pass approach was used to avoid in-plane loss limitation [23]. This velocity boundary synchronized with the heart movement defined by atrial contraction and relaxation. The composition of zebrafish blood was assumed to be similar to that of humans, and viscosity was estimated from the relative viscosity and particle volume fraction using literature data [2,24]. The obtained value, 7 centipoise (cP), was close to that of the trout (8cP) [20]. The density of blood was assumed to be similar to that of humans (1.06g/cm^2^).

### Moving domain CFD simulation

The individual developmental stages were captured and the video frames were extracted by ImageJ (National Institute of Health, Bethesda, MD, USA). A custom program written in Matlab was used to create two dimensional segmentations and meshes from the recorded images. For the individual images, the program allows the user to select points on the image to trace the boundary of the endocardium. Spline curves were generated from the manual boundaries, from which equidistant points were created to define nodes on the segmentation. For each set of data, a constant number of nodes were created in the segmentation of the endocardial wall in each individual image frame, allowing for easy Lagrangian tracking of the heart wall motion. This process creates segmentations to describe the wall movements with a temporal resolution similar to the resolution of the image acquisition. The movements of the nodes in the segmentations were then interpolated over time using spline functions, producing wall motion data at high temporal resolution for the simulation. The frame rate of recorded video was 20 frames per second (fps), and the wall motion for three cardiac cycles was re-analyzed into 100 time points per second to enable smooth wall motion prescription.

For each simulation, a two dimensional unstructured mesh was generated using a Delaunay triangulation algorithm from the segmentation representing the heart at its most contracted time point. Based on the wall motion described by the segmentations over the cardiac cycle, the mesh was then deformed accordingly during the CFD simulation as described below (Code S1).

An in-house finite element model was employed, assuming an incompressible and Newtonian fluid. The computational domain, Ω = Ω(t), moved with prescribed wall motion. Hence, an arbitrary Lagrangian Eulerian approach was used to formulate this problem [25]. In this framework, the Navier-Stokes equations are as follows:

$$\rho \dot{u}+\rho (u-\tilde{u})\cdot \nabla u-\nabla \cdot T=0,\;$$ (1)

∇⋅u=0, (2)

$$T=-pI+\mu (\nabla u+\nabla u^{T}),$$ (3)

$$u=g,\;\;\;\;x=\Gamma^{g}$$ (4)

$$T\cdot n=h,\;\;\;\;x\in \Gamma^{h}$$ (5)

Where *ρ* denotes the density, $\dot{u}$=$\dot{u}\text{​}\text{​}\text{​}\text{​}\;(x,t)$ represents the velocity time derivative acquired with respect to a fixed spatial location, *u*=*u* ​​​​(*x*,*t*), the fluid velocity vector, $\tilde{u}$=$\tilde{u}\text{​}\text{​}\text{​}\text{​}\;(x,t)$, the mesh velocity (spatial domain velocity), *p*=*p*​​​​ (*x*,*t*), pressure, and *T*, the stress tensor. In Equations (4) and (5), the Neumann and Dirichlet boundaries are denoted by *Γ* ^*h*^ and *Γ* ^*g*^, respectively. A no-slip boundary condition was assumed, and *g*= *g*​​​​ (*x*,*t*) described the prescribed motion based on the image data. Assuming zero traction for the outlets, we imposed *h*= 0 at the outlets and an essential (Dirichlet) boundary condition at the inlet. Due to the presence of reversed flow at the outlets, special care was taken to avoid rapid simulation divergence by using a minimally intrusive method, in which an advective stabilization term was added to the weak form [9].

In the discrete setting, a stabilized formulation [26–29] was used to allow for equal-order velocity and pressure interpolation, and for addressing the convective instability associated with Galerkin’s method. The second order generalized-α method for the time integration [30], linear finite elements in space, and a modified Newton-Raphson method is used for linearization of Equations (1) and (2). The linear system of equations was then solved by forming the Schur compliment and by using a combination of GMRES [31,32] and conjugate gradients methods.

Using a Quasi-direct coupling method [33], after solving Equation (1) and Equation (2) and obtaining the velocity and pressure for the entire domain, a linear elasticity equation was solved to capture the mesh motion. This was accomplished in an iterative loop to ensure simultaneous convergence of both equations. To preserve mesh quality, we used the Jacobian stiffening procedure, in which the mesh Young’s modulus was inversely proportional to the determinant of the element Jacobian [34].

The simulation was run for 5 seconds of physical time, the run-time of the video, with a time step size of 2 ms. The non-linear iterations were continued until the norm of the residual vector reduced below 0.001 or the number of iterations exceeded 5. After simulation, the post-processing tool Paraview (Clifton Park, NY, USA) was used to visualize the calculated intracardiac shear stress, the pressure gradient between the atrium and ventricle, and velocities past the AV canal.

### Calculating Parameters

Averaged WSS was obtained from the nodes near the AV canal. The region of interest was specified by cropping near the AV canal, averaging the values at the highest 10 nodes. Shear stress is defined as velocity gradient multiplied by dynamic viscosity (μ) as follows:

$$\tau =\mu \frac{du(y)}{dy}$$ (6)

where μ is the dynamic viscosity of blood of the embryonic zebrafish.

Since exact pressure was not provided as a boundary condition, pressure gradient was calculated. Nodes in the center of both chambers were selected to compare the pressure value. To avoid error, 10 points were averaged at the location of maximum pressure.

To plot the trajectory of blood particles with instantaneous streamlines, stream functions were used. The two-dimensional stream function is defined:

u=∇×ψ (7)

where ψ=(0,0,ψ), and $u=\left(u_{x},u_{y},0\right)$, which in 2D is:

$$u_{x}=\frac{\partial \psi }{\partial y}{\mathrm{, u}}_{y}=-\frac{\partial \psi }{\partial x}$$

The ventricular ejection fraction (VEF) was estimated on the assumption of an ellipsoidal bipolar shape [1,35,36] as follows:

$$V=\frac{\pi }{6}L\;D_{1}D_{2}$$ (8)

where *V* denotes the ventricular volume, *L* is the longest length of the ventricle, and D_1_ and D_2_ are the orthogonal minor diameters of the ventricle [35,36]. Assuming that D_1_ and D_2_ are equal, we applied the VEF equation:

$$VEF=\frac{Stoke\;\;Volume}{End\;\;Diastolic\;\;Volume}=\frac{End\;\;DiastolicVolume\;\;-\;\;End\;\;Systolic\;\;Volume}{End\;\;Diastolic\;\;Volume}$$ (9)

The Reynolds number at the AV canal and the Womersley number at the inflow tract were calculated based on following equations:

Re=ρuDμ (10)

$$\alpha^{2}=\frac{\rho \omega R^{2}}{\mu }$$ (11)

where D is the diameter of the AV canal, ω is the angular frequency, and R is the radius of the inflow tract.

### Statistical Analysis

All values will be expressed as means ± SD. For statistical comparisons of averaged peak shear stress and pressure gradients, we used a paired *t*-test with values of *P* < 0.01for WSS and pressure gradients considered as significant. A comparison of multiple mean values was performed by one-way analysis of variance (ANOVA), and statistical significance among multiple groups was determined using the Tukey procedure.

## Results

### Validation of CFD with PIV at different developmental stages

**Figure 3:**
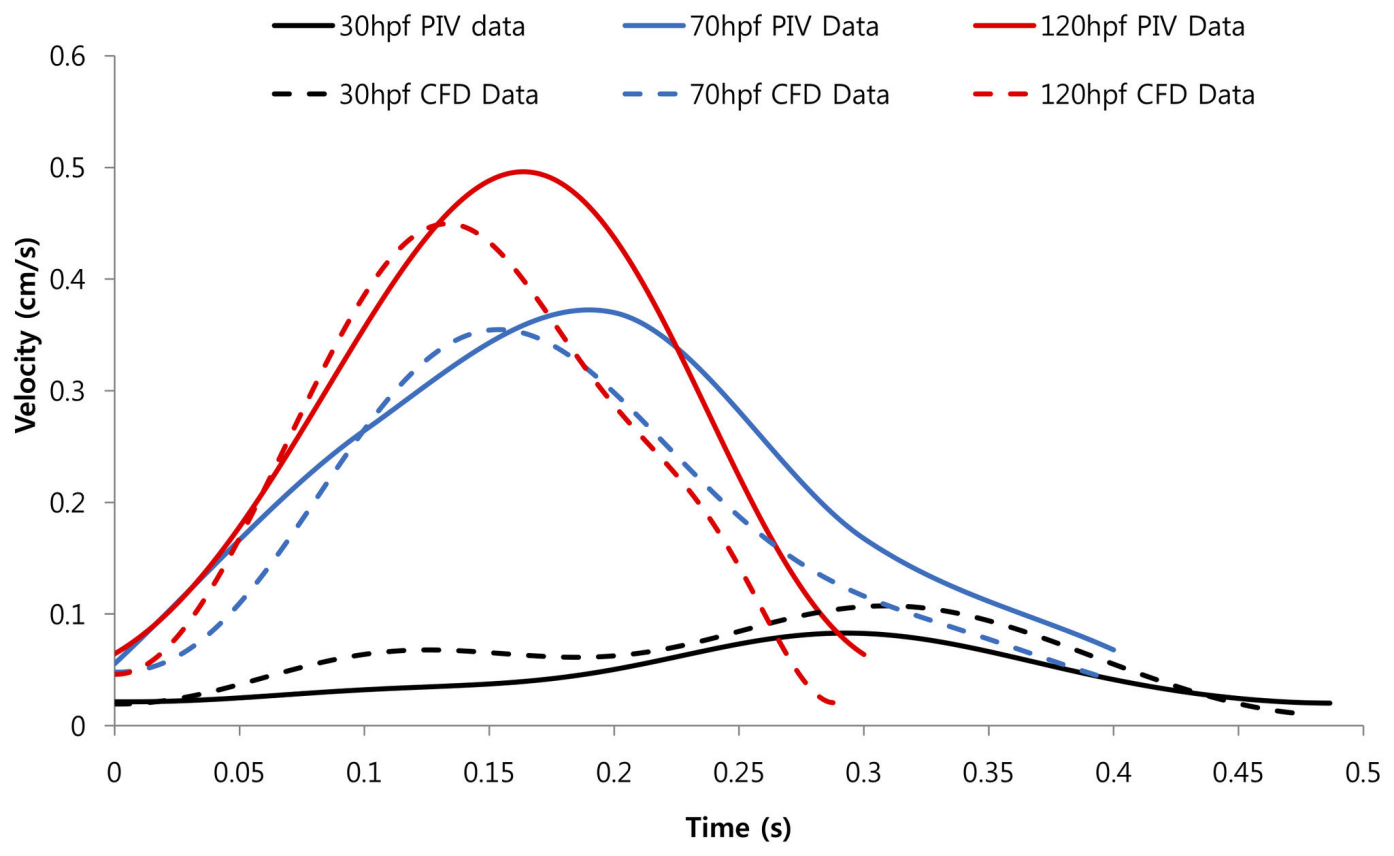
CFD simulation data was obtained at AV canal at three different developmental stages; namely, 20-30 hpf, 60-70 hpf, and 110-120 hpf. The velocity profiles were validated by comparing with those of particle image velocimetry (PIV) via the Matlab-coded PIV tool. The CFD simulations overlapped reasonably with those of PIV. Noticeable are the increase in both the blood velocities and heart rates at later stages in development.

To validate the time-dependent CFD code, we compared with the Matlab derived-particle image velocimetry (PIV) data at the AV canal. The average velocity over the cross section during atrial contraction closely overlaps with those of CFD during the upstroke of atrial systole at 20-30 hpf, 60-70 hpf, and 110-120 hpf, respectively, despite moderate underestimation during the down stroke (Figure 3). While both velocities and heart rates increase, the duration of atrial systole shortens from the early to later developmental stages (Table 1). The CFD and PIV provide a reasonable agreement to investigate the dynamics in physiological parameters during cardiac morphogenesis.

**Table 1:** Hemodynamic parameters.

	30hpf	50hpf	70hpf	90hpf	120hpf
Heart Rate (bpm)	54±2	75±16	92±36	117±25	149±20
Averaged VEF(%)	76±10.6	65±7.2	59±5.0	65±6.6	63±3.9
*Re* ^*^ at AV canal when atrium contract	0.07±0.03	0.09±0.03	0.13±0.02	0.17±0.03	0.23±0.07
α^#^ at inflow tract of the heart	0.11±0.01	0.20±0.01	0.13±0.00	0.14±0.01	0.13±0.01

^*^ Reynolds number, ^#^ Womersley number

### Spatial and temporal variations in wall shear stress (WSS) and velocity profiles

**Figure 4:**
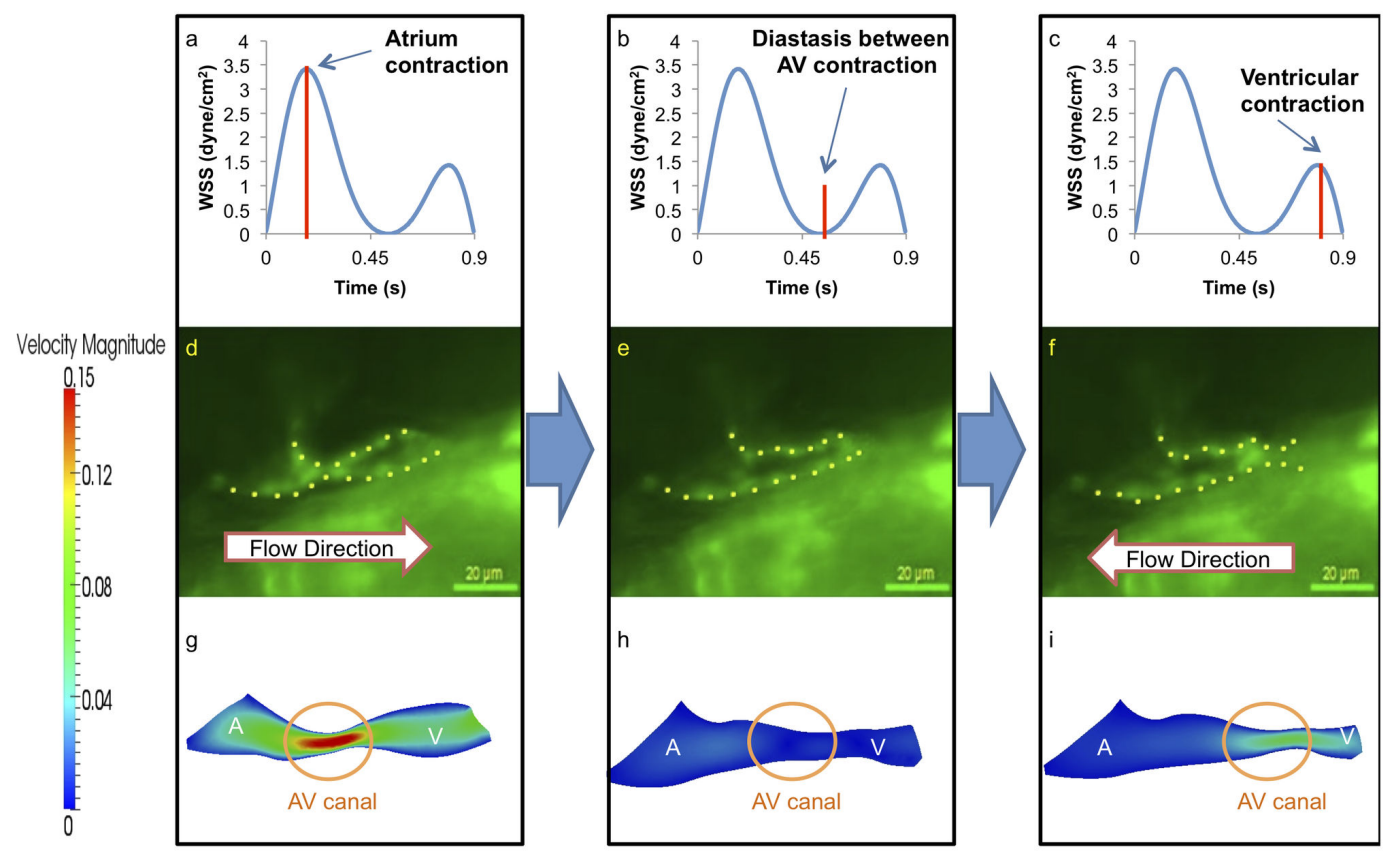
At 20-30 hpf, zebrafish heart is morphologically a tube-like structure with a AV canal (AV) separating the atrium (A) and ventricle (V). (a) Atrial contraction, the first peak, engenders an increase in shear stress profiles through the AV canal. (b) Diastasis between atrial and ventricular contraction result in a decrease in shear stress across the AV canal. (c) Ventricular contraction, the second peak, results in flow reversal or regurgitation through the AV canal. The magnitude of wall shear stress (WSS) across the AV canal is about half of the forward blood flow at AV canal. (d) The green fluorescent protein (GFP) delineated the tubular structure. Unidirectional forward flow from the contracting atrium into the ventricle develops through the AV canal. (e) The GFP image reveals movement of peristaltic wave to the end of tubular heart structure. (f) Flow reversal develops in response to the contraction of tubular structure. (g) Velocity profiles show the maximal magnitude through the AV canal. (h) The magnitude of velocity is minimal at AV canal. (i) The magnitude of flow reversal or regurgitation is about half of the forward blood flow at AV canal. Contoured velocity profiles confirm the observed result (g–i). Red bars in (a–c) represent the time points at which the corresponding velocity profiles were reconstructed. A, atrium; V, ventricle.

CFD simulations were compared at five developmental stages from 20–30 hpf to 110-120 hpf. At 20-30 hpf, peristalsis of a tube-like structure begins with atrial contraction, followed by ventricular contraction. The AV canal oscillates moderately in relation to atrial and ventricular diastole. Atrial systole produces forward flow, while ventricular systole results in reversal flow or regurgitation across the AV canal (Figure 4a–c). The second peak of WSS magnitude reflects flow regurgitation at the AV canal during atrial diastole, with a magnitude of about half that of forward flow. The green fluorescent protein (GFP)-labeled tubular heart structure is visualized and the direction of blood flow is indicated (Figure 4d–f). CFD highlights spatial and temporal variations in velocity profiles (Figure 4g–i). During diastasis, velocity through the AV canal is minimal. Furthermore, there is peristaltic atrium motion during systole from the inflow tract to the AV canal. At the end of peristaltic wave, contraction of tubular structure induced the flow reversal through the AV canal. The following wave from the inflow tract prevents flow reversal into the inflow tract from the atrium chamber [3,16], though the ventricular motion is simple contraction.

**Figure 5:**
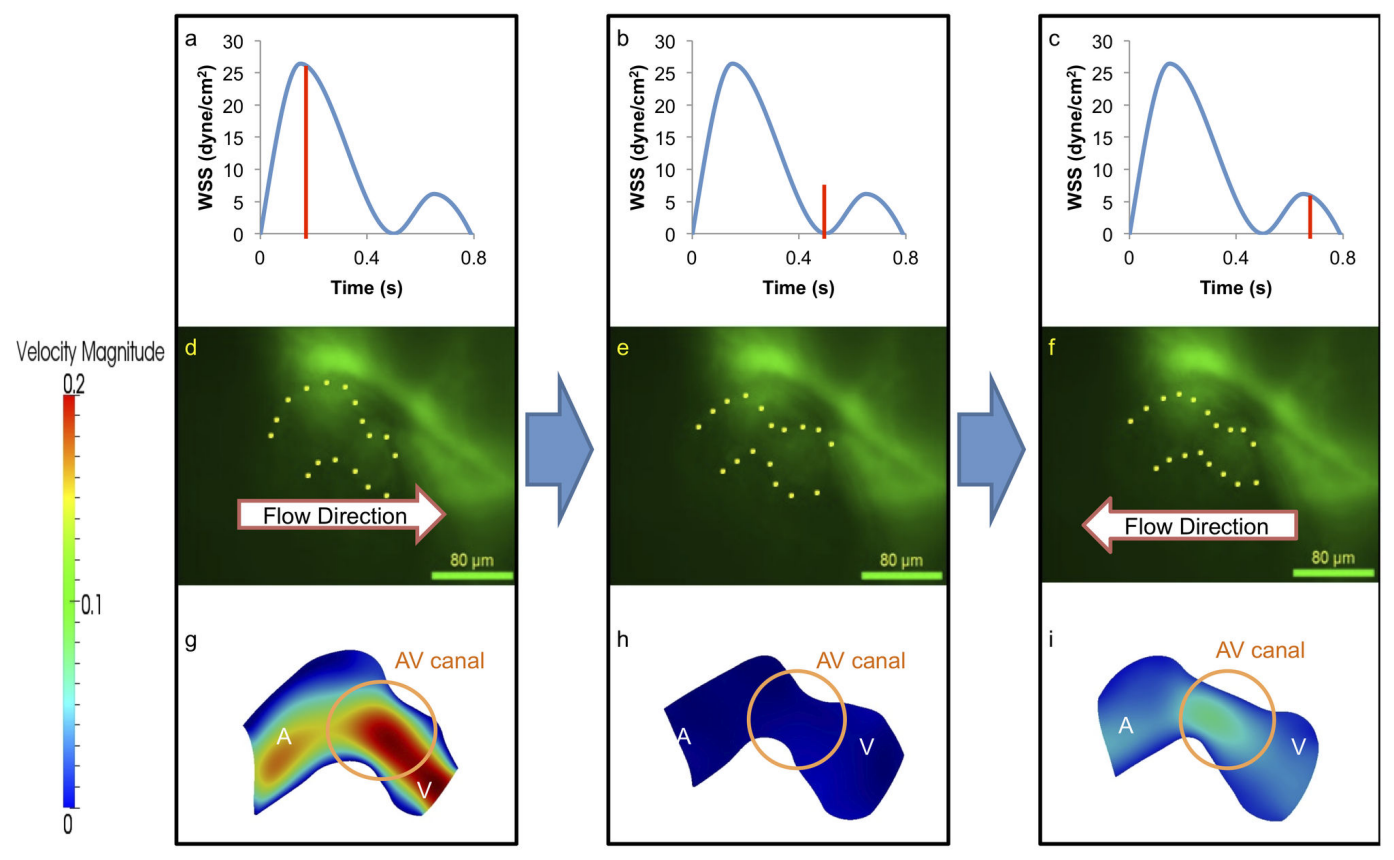
At 40-50 hpf, the zebrafish heart begins to undergo looping. (a–c) Compared to 20-30 hpf, WSS across the AV canal is increased by ~9-fold during atrial contraction. However, the magnitude of flow reversal or regurgitation-induced WSS during ventricular contraction decreases by ~33% in comparison with the earlier stages as the AV canal is developing into a valvular structure. (d–i) The ventricle has increased in size when compared to the atrium and absolute values of velocity have increased by 33%, A, atrium; V, ventricle.

At 40-50 hpf, the tube-like structure undergoes cardiac looping, accompanied by a nearly 9-fold increase in the magnitude of peak WSS during atrial systole, but a 3-fold reduction during ventricular systole compared to atrial systole (Figure 5a–c). Cardiac looping is associated with diminishing flow regurgitation (Figure 5d–f). At this stage, the morphological ventricle increases in diameter in comparison with the atrium, and absolute values of peak velocity also increase by approximately 33% (Figure 5g–i).

**Figure 6:**
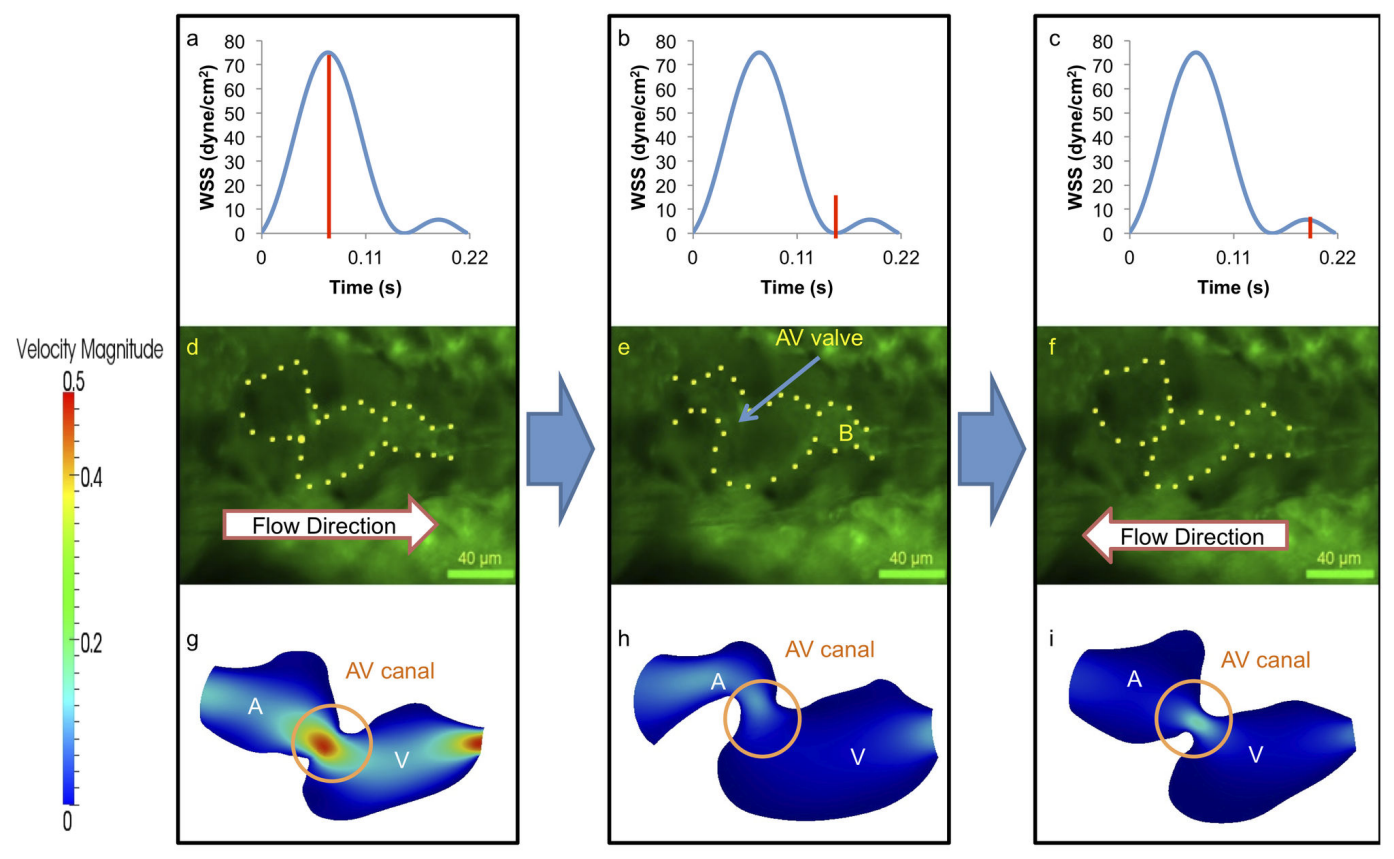
At 110-120 hpf, the morphologic AV valve is distinct and left ventricle is enlarging. (a–c) WSS across AV valve in response to atrium contraction is significantly higher than that of ventricular contraction. (d–i) Complete formation of the AV valve and bulbus arteriosus was observed at this stage, and amount of flow reversal reduced. The ventricle has become larger than the atrium in size, and the flow reversal through the AV canal during ventricular contraction is reduced due to small size of AV canal. A, atrium; V, ventricle; B, bulbus arteriosus.

At 110-120 hpf, peak systolic WSS increases by nearly 20-fold compared to early stage values at 20-30 hpf during atrial contraction, while peak diastolic WSS induced by regurgitation further diminishes in the presence of the morphological AV valve (Figure 6a–c). At this stage, the AV valve leaflets and bulbus arteriosus adjacent to the ventricle develop, resulting in reduced velocity magnitude during atrial diastole (Figure 6d–f). Furthermore, spatial and temporal variations in the velocity profiles reveal reduction in the magnitude of backward flow compared to forward flow (Figure 6g–i). Thus, these hemodynamic findings are consistent with physiologic changes during heart development.

### Linking hemodynamic parameters with cardiac morphogenesis

**Figure 7:**
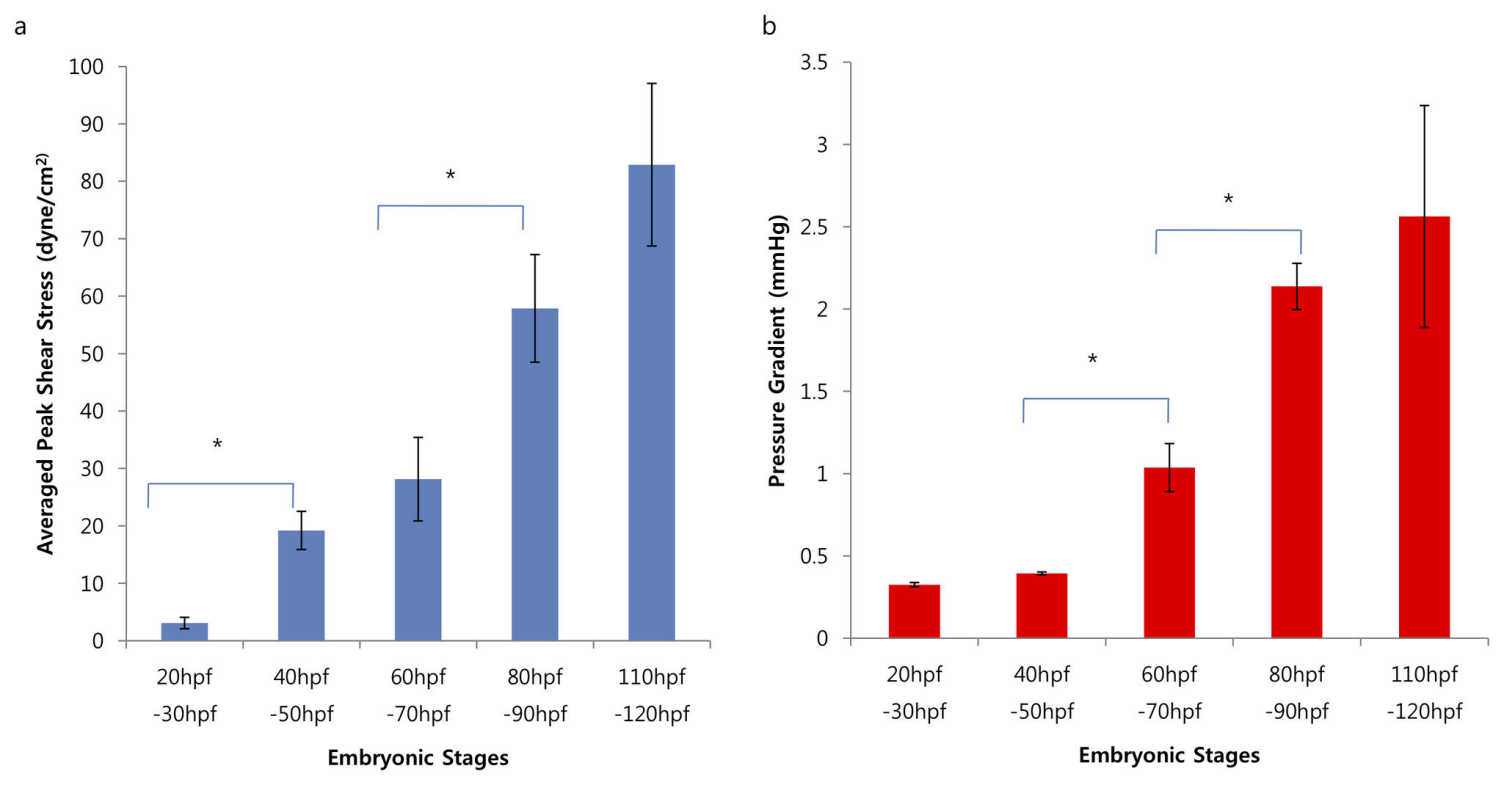
Statistical data for average peak shear stress and pressure gradient. (a) Average peak shear stress increases during developmental stages. At 50 hpf, the heart has undergone cardiac looping, which corresponds to an increase in magnitude of shear stress by 5-fold from 30 to 50 hpf (^*^ *p* < 0.01, n = 6) (b). In corollary, average peak pressure gradient across morphological AV canal increased from early to later developmental stages (^*^ *p* < 0.01, n = 6).

We assessed changes in mean peak shear stress (MPSS), pressure gradient (∇*P*), and vorticity fields in relation to structural changes. MPSS at the AV canal increases by 5-fold from 20–30 hpf to 40-50 hpf, during which cardiac looping occurs (*p* < 0.01, n = 6) (Figure 7a). MPSS further increases by 2-fold from 60–70 hpf to 80-90 hpf (*p* < 0.01, n = 6), during which the AV cushion starts to develop into a morphological AV valve [37,38] (Figure 7a). The pressure gradient (∇*P*) across the AV canal also increases significantly from 40–50 hpf to 60-70 hpf and from 60–70 hpf to 80-90 hpf (Figure 7b) (*p* < 0.01, n = 6), during which trabeculation is forming in the ventricle [5].

### Development of ventricular vortices during chamber morphogenesis

**Figure 8:**
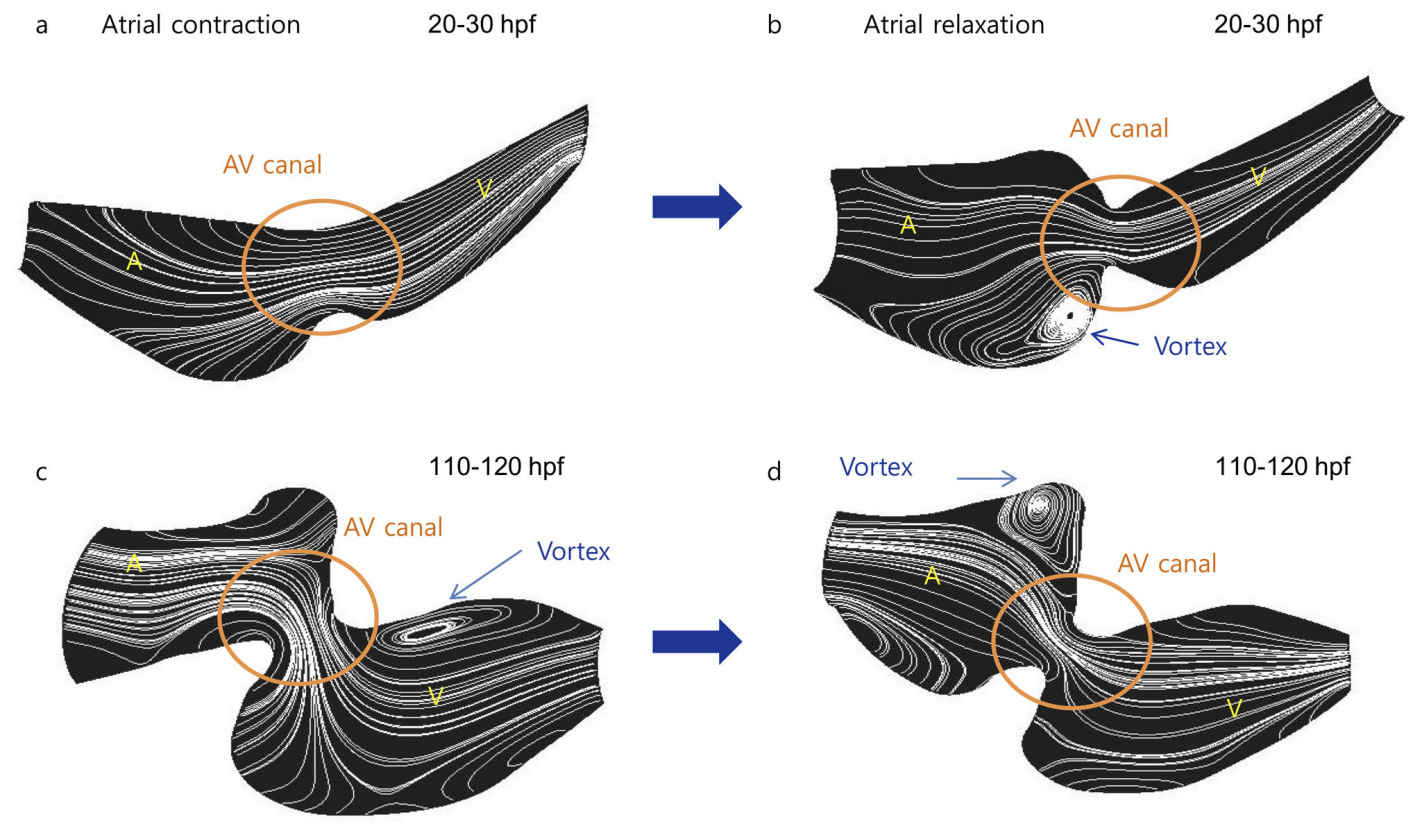
Instantaneous streamlines from early to the later stages. (A–B) At the early stage (20hpf-30hpf), vortices in the morphological atrium is present in response to flow reversal during contraction of the morphological ventricle. (C–D) At 110 to 120 hpf, vortices are present downstream from the AV valve during atrial contraction and upstream (in the atrium) during ventricular contraction.

Vorticity fields and instantaneous flow streamlines reveal laminar flow profiles occurring in both early (20-30 hpf) and later stages (110-120 hpf) (Figure 8). At 20-30 hpf, unsteady vortex features develop during atrial relaxation (Figure 8b). At 110-120 hfp, unsteady vortices develop in both the atrium and ventricle (Figure 8c and d). While the mechano-biological implication of disturbed or vortical flow remains to be established, trabeculation, highly organized sheets of cardiomyocytes forming muscular ridges, was observed during later developmental stages, suggesting clinical relevance for future investigation [5].

Furthermore, we compared changes in heart rates, ventricular ejection fraction, Reynolds, and Womersley numbers (Table 1). Heart rates steadily rose while ventricular ejection fraction remained relatively unchanged between 60% and 65% at later stages of development. Despite incremental increase, the Reynolds numbers at the AV canal remained less than 0.23 consistent with laminar flow. The Womersley numbers at the inflow tract also remained less than 0.13, consistent with parabolic inlet velocity profiles. Taken together, the moving boundary CFD modeling provides a hemodynamic basis to elucidate mechano-transduction underlying cardiac morphogenesis.

## Discussion

While direct measurement of intracardiac properties remains an unmet bioengineering challenge, the advent of CFD analysis is conducive to linking hemodynamics with cardiac morphogenesis. While WSS clearly impacts genetic programming of the developmental heart, there is a paucity of methodologies for assessing shear stress in real-time in small animal models of heart development. Thus, the novelty of our approach lies in the reconstruction of time-dependent moving endocardial boundaries and interpolation between measurements using *in vivo* imaging of the zebrafish heart.

In this proof-of-concept study, we applied two-dimensional simulations together with advanced imaging of the zebrafish heart. *Tg*(*fli1a:EGFP*)^*y1*^ zebrafish has been used in angiogenesis studies due to their optically translucent green fluorescent endothelial cells [39]. The use of *Tg*(*fli1a:EGFP*)^*y1*^ transgenic embryos allows for clear delineation of the inner wall for reconstructing the moving boundary for CFD. These genetically engineered zebrafish embryos express green fluorescent protein (GFP)-labeled *fli1*a gene in endothelial cells lining the endocardium and blood vessels. Treatments with PTU beginning at 10 hpf further prevents pigmentation, enhancing organ visualization. To reconstruct the two-dimensional CFD models, we focused on the mid-plane of the atrium and AV valve where milestones in intracardiac development occur in relation to blood flow across the AV canal. Recorded video was extracted frame-by-frame to track the motion of the beating heart. Wall motion was generated by the interpolation between frames. The inlet velocity boundary condition from PIV was employed in the simulation.

Comparing the average peak shear stress at various stages revealed key milestones of cardiac morphogenesis. A significant increase in peak shear stress correlated with cardiac looping at 40-50 hpf (Figure 7a), during which shear stress on the endothelial and endocardial inner lining of the vascular system will be transmitted either intracellularly, resulting in changes in gene expression, e.g., *Krüppel-like* factor-*2* (*klf2a*) [40], or trans-epithelially, leading to signaling the underlying mesenchyme or myocardium [41]. Furthermore, the significant increase in shear stress between 60-70hpf and 80-90 hpf is implicated in endocardiac cushion formation, which is a precursor to AV valve formation [37,42], and is associated with up-regulation of *klf2a* mRNA [2] as well as Notch, NFAT, ErbB and Tgf-β signaling [4,37,38,43].

Clinically, left ventricular non-compacted cardiomyopathy is associated with a dense ventricular trabeculations, and thus, reduced cardiac systolic function [44]. In humans, trabeculae form as the ventricular myocardium protrudes into the lumen of the chamber, resulting in increasing muscle mass and altered cardiac output. In zebrafish embryos, trabeculation is not evident before 48 hpf, and trabeculation starts at the end of cardiac looping [6,17]. Prior to trabeculation, the inner surface of the heart is smooth and the cardiomyocytes have uniform thickness [6]. In the current study, CFD simulation unravels that the incremental increase in pressure gradient across the AV canal prior to 50 hpf is relatively low compared to the later developmental stages (Figure 7b). However, a significant increase in pressure gradient coincides with trabeculation after 50 hpf-90 hpf, during which trabeculation ridges, bundles, and stalks thicken and begin to protrude into the ventricle [6]. In this context, significant changes in pressure gradient may be implicated in the initiation of trebaculation. However, further investigation is warranted to address changes in pressure gradient versus shear stress as the potential mechanism underlying the formation of trabeculation.

Vortices induced by blood flow are also implicated in remodeling of the left ventricle [45]. The velocity field and instantaneous streamlines reveal structures occurring during atrial diasotle at the early stage of the tube-like structure (Figure 8). When the diameter of the AV canal is reduced and the morphological AV valve forms, vortices are present in both the atrium and ventricle during both atrial systole and diastole. Despite the relative small size of vortical structures based on the vorticity fields (Figure 8), implications in mechano-signal transduction from endocardium to myocardium warrant further investigation.

PIV provided validation to changes in velocity across the AV canal at three different developmental stages. The findings support the physiological changes in heart rates in relation to the increase in blood velocity. At 120 hpf, the heart rate was in the range of 120 and 180 bpm, approaching those of adult zebrafish (Table 1) [46]. We further estimated ventricular ejection fraction (VEF) as a global approach to assess cardiac performance [21]. At early embryonic stages, VEF varies considerably (n=6), and this value was close to that of humans [47]. Reynolds numbers, defined as a ratio of inertial forces to viscous forces [16,48], are less than 1, and Wormersley numbers, defined as a ratio of transient inertial forces to viscous forces [48], are also less than 1. Together with vorticity fields and instantaneous streamlines (Figure 8), laminar flow was observed in the zebrafish embryonic heart.

The limitations of the current study lie in the use of PTU and the slow frame rate for 2-D CFD simulations. Treatment of embryos with PTU to prevent pigmentation is reported to delay development, and to promote cardiac developmental defects in some embryos [49]. While PIV validates the CFD analysis, PIV focuses solely the movement of the particles, whereas the CFD code requires additional physical parameters, including the moving boundary, inlet velocity, and heart rate. Approximation of structure by a heart mid-plane analysis also posed a challenge for non-planar anatomy among atrium, ventricle, inflow tract, outflow tract, and AV canal. Particularly, during cardiac looping, especially, the image acquiring among two chambers, inflow tract, and outflow tract is challenging in the plane. The low frame rate may have underestimated wall deformation after interpolation between frames. Higher pixel binning of the microscope would increase both signal to noise (SNR) and temporal resolution (frame rate) despite a decrease in spatial resolution. However, increasing the magnification of the lens coupled with increasing binning would hold promise to enhance both temporal and spatial resolution [50,51].

In summary, our moving domain CFD simulation provides a quantitative basis to link hemodynamics with cardiac morphogenesis; namely, cardiac looping, AV canal and endocardial formation, and future study of trabeculation. The 2-D simulation data paves the way to further develop 3-D CFD models of heart development, coupled with the 3-D moving boundary data from customized optical imaging techniques [52,53]. The translational implication of our study is to elucidate mechano-signal transduction with clinical relevance to congenital heart disease and left ventricular non-compacted cardiomyopathy.

## Supporting Information

Video S1 **Zebrafish heart development from 20–30 to 110-120 hpf.** The tubular shape of the heart was first observed from 20–30 hpf. At 40-50 hpf, the heart is undergoing looping. The atrium and ventricle are fully formed at 60-70 hpf. The AV cushion begins to develop at 80-90 hpf, and becomes a functional AV valve at 110-120 hpf.
(MOV)

Video S2 **Blood particles visualization from 110-120hpf.** *Tg(gata1:dsRed)sd2* transgenic zebrafish allowed visualization of blood particles using red fluorescence. Particle tracing provided velocity information from the inflow tract.
(MOV)

Code S1 **Segmentation program for zebrafish heart images.** This code is used to segment images of the zebrafish heart. Nodal points of the wall and outlets are created to allow for 2-D simulations, and all necessary information must be entered in the program. The program files must be placed in the same folder as the files for the images to be segmented. The user defines wall boundaries by picking points via "clicking" and finalizing by pressing "enter." Define the wall boundaries starting at the inlet and ending at the outlet, repeating twice for each image to define both sides of the wall. This program is used when there is both an inlet and outlet which need to be simulated.
(ZIP)
